# Telemedical Direction to Optimize Resource Utilization in a Rural Emergency Medical Services System

**DOI:** 10.5811/westjem.18427

**Published:** 2024-08-27

**Authors:** Ramesh Karra, Amber D. Rice, Aileen Hardcastle, Justin V. Lara, Adrienne Hollen, Melody Glenn, Rachel Munn, Philipp Hannan, Brittany Arcaris, Daniel Derksen, Daniel W. Spaite, Joshua B. Gaither

**Affiliations:** * The University of Arizona, College of Medicine, Department of Emergency Medicine, Tucson, Arizona; † The University of Arizona, College of Medicine, Department of Emergency Medicine, Phoenix, Arizona

## Abstract

**Background:**

Telemedicine remains an underused tool in rural emergency medical servces (EMS) systems. Rural emergency medical technicians (EMT) and paramedics cite concerns that telemedicine could increase Advanced Life Support (ALS) transports, extend on-scene times, and face challenges related to connectivity as barriers to implementation. Our aim in this project was to implement a telemedicine system in a rural EMS setting and assess the impact of telemedicine on EMS management of patients with chest pain while evaluating some of the perceived barriers.

**Methods:**

This study was a mixed-methods, retrospective review of quality assurance data collected prior to and after implementation of a telemedicine program targeting patients with chest pain. We compared quantitative data from the 12-month pre-implementation phase to data from 15 months post-implementation. Patients were included if they had a chief complaint of chest pain or a 12-lead electrocardiogram had been obtained. The primary outcome was the rate of ALS transport before and after program implementation. Secondary outcomes included EMS call response times and EMS agency performance on quality improvement benchmarks. Qualitative data were also collected after each telemedicine encounter to evaluate paramedic/EMT and EMS physician perception of call quality.

**Results:**

The telemedicine pilot project was implemented in September 2020. Overall, there were 58 successful encounters. For this analysis, we included 38 patients in both the pre-implementation period (September 9, 2019–September 10, 2020) and the post-implementation period (September 11, 2020–December 5, 2021). Among this population, the ALS transport rate was 42% before and 45% after implementation (odds ratio 1.11; 95% confidence interval 0.45–2.76). The EMS median out-of-service times were 47 minutes before, and 33 minutes after (*P* = 0.07). Overall, 64% of paramedics/EMTs and 89% of EMS physicians rated the telemedicine call quality as “good.”

**Conclusion:**

In this rural EMS system, a telehealth platform was successfully used to connect paramedics/EMTs to board-certified EMS physicians over a 15-month period. Telemedicine use did not alter rates of ALS transports and did not increase on-scene time. The majority of paramedics/EMTs and EMS physicians rated the quality of the telemedicine connection as “good.”

Population Health Research Capsule What do we already know about this issue?

*Telemedicine remains underused in rural EMS systems, with concerns about increasing ALS transports, extending on-scene times, and connectivity issues.*

What was the research question?

*How does implementing telemedicine in rural EMS affect patient management and system performance for chest pain cases?*

What was the major finding of the study?

*Telemedicine did not change ALS transport rates (42 vs. 45%, OR 1.11, 95% CI 0.45–2.76) or increase on-scene times (47 vs. 33 minutes, p = 0.07), and 64% of EMS staff rated call quality good.*

How does this improve population health?

*Telemedicine allows rural EMS to maintain ALS availability, reducing strain on limited resources and potentially improving outcomes for chest pain patients.*

## INTRODUCTION

Telemedicine has improved healthcare delivery and outcomes for rural populations.^1^ ^–^ ^5^ As rural communities across the United States (US) struggle to recruit, train, and retain paramedics and emergency medical technicians (EMT), these commuities are left with a shortage of qualified individuals to provide healthcare and an increased cost to deliver that care.^6^ ^–^ ^9^ Telemedicine for emergency medical services (EMS) may be particularly useful in rural communities that face paramedic shortages.^2^ ^–^ ^4^

With paramedic shortages, many rural EMS agencies often depend on a lone paramedic to serve their community. In this setting, when two 911 calls overlap, the rural community is left without Advanced Life Support (ALS) coverage. This gap in ALS service is particularly important in the care of patients with chest pain. For example, assume an ALS transport is needed in a rural community and the sole paramedic is taken out of the service area for hours. Then assume that during that same period, a second 911 call for chest pain occurs. For that second call, the community is left with only a Basic Life Support (BLS) responder who cannot interpret electrocardiograms (ECG). In this scenario either a helicopter is called, increasing cost of service delivery, or the patient is transported emergently by BLS responders, increasing risk to both the EMTs and the patient. Clearly, in this scenario there is the potential for a telemedicine physician to reduce some of the burden placed on resource-limited communities.

However, we found that some EMS systems are reluctant to implement these programs for a variety of reasons. Paramedics shared concerns that a physician’s policy of mandating ALS transport for all patients might lower the physician’s liability at the expense of increasing the number of ALS-required transports. Others were concerned that performing a telemedicine visit would take a significant amount of time, thus further reducing availability of ALS resources. Finally, there were also some concerns about the lack of access to cellular data and whether the telemedical solution would be available when needed.

These potential barriers are important to evaluate prior to widespread implementation of EMS telemedicine solutions. To evaluate the benefits and potential risks, a rural EMS telemedicine pilot project was implemented targeting patients with chest pain. Throughout that pilot project, program partners collected quality improvement (QI) data to ensure that the telemedicine program was functioning as designed and did not adversely affect system performance. Our aim in this analysis was to evaluate quality data points and assess the impact of telemedicine on EMS management of patients with chest pain; we also evaluated perceived barriers by EMS staff.

## METHODS

### Study Design

This study was a retrospective review of data collected by a single EMS agency throughout the project. We collected data for the primary purpose of QI and evaluation of the telemedicine platform. STROBE methodology was used.^10^ We used two datasets for this retrospective review. Firstly, we looked at quantitative data from a prehospital QI dataset in which a 12-lead ECG was performed. Secondly, we analyzed the primary chief complaint of chest pain. Cases from that QI dataset were included in this analysis if they occurred during the 12-month pre-implementation study period or the 15-month post-implementation period. We also evaluated qualitative data from a second QI dataset that was completed by paramedics/EMTs and EMS physicians after each telemedicine platform use. That dataset contained the paramedic/EMT and EMS physicians’ subjective evaluation of the telemedicine call quality.

### Study Setting

The AzQuality project served as a pilot initiative designed to establish a telemedicine service, enabling rural paramedics and EMTs to access urban medical resources during emergency care for patients experiencing chest pain who dialed 911. The EMS telemedicine pilot was implemented at a single, rural EMS agency. At the time of introduction and during new employee orientation, both EMTs and paramedics were taught how to use the telemedicine system via a brief lecture and a hands-on practice session. After implementation, EMTs and paramedics were instructed to use the Telemedicine tool to contact board-certified EMS physicians for patients experiencing chest pain 24 hours a day, 7 days a week. They could also use the tool for other encounters as needed. Telemedicine services were provided by board-certified EMS physicians from a single, large EMS physician group.

The telemedicine pilot program was implemented by the Sonoita-Elgin Fire District, a rural EMS agency and sole 911 responding agency for a large, geographically diverse area in Southeastern Arizona. The GD “e-Bridge” communication platform (General Devices LLC, Ridgefield, NJ) was selected by the program leadership group as the telemedicine platform for this pilot program. This software allowed paramedics/EMTs to conduct the telemedicine visit as well as transmit photos of ECGs or other clinical data to the EMS physicians. The e-Bridge system was operated on commercially available smartphones.

Prior to and during the pilot project, when available, paramedics responded to all 911 calls. When unavailable, BLS crews would respond alone. Both paramedics and EMTs were asked to use the telemedicine system any time they encountered a patient with chest pain. After EMT or paramedic patient assessment, the EMS physician was contacted via e-Bridge and given a brief patient presentation. The EMS physician had access to the patient’s vital signs and 12-lead ECG through the e-Bridge software. After a brief interaction with the patient, the physician risk-stratified patients as low, moderate, or high risk for adverse events during transport to definitive care. The paramedics/EMTs were then given online medical direction for ground transport by BLS for low-risk patients, ALS ground transport for moderate-risk patients and helicopter EMS transport (HEMS) of patients who were felt to be at high risk for adverse events during transport.

### Outcome Measures

The primary outcome of this retrospective review was the rate of BLS, ALS, and HEMS transport. Secondary outcomes (Table 1) included EMS system service delivery times and subjective evaluation of how well the interaction went (good, fair, poor). The EMS service delivery times were collected from EMS agency computer-aided dispatch systems. Subjective data on the overall system performance and telemedicine platform call quality were collected and managed using Research Electronic Data Capture (REDCap) tools hosted at the University of Arizona. REDCap is a secure, web-based software platform.^11^ ^,^ ^12^ A survey was also launched immediately after each telemedicine encounter. Basic call data (data and time) was then used to link the subjective telemedicine platform evaluation to EMS call data.

**Table 1.** Secondary outcomes in study of telemedicine use by first responders in rural Arizona.

Secondary outcome	Definition
Total unit out-of-service time	Time from dispatch to when the transporting unit becomes available.
Response time	The interval from dispatch to arrival on scene.
On-scene time	Duration from arrival on scene to either the initiation of transport or the point at which the patient refuses transport or the unit becomes available without transport.
Transport time	Time from the initiation of transport to when the unit is again available.
Responder/clinician experience	Subjective experience of paramedics/EMTs and EMS physicians using the telemedicine system rates as poor, fair, or good.

*EMT*, emergency medical technician; *EMS*, emergency medical services.

### Data Analysis, Regulatory Approval and Role of Funding

The EMS agency and its medical director provided deidentified QI data for the purpose of review for this analysis. Simple descriptive statistics summarized the results. The University of Arizona Institutional Review Board approved the review of this project’s QI data analysis and publication. The Rural Health EMS Flex Supplement grant from the federal Health Resources and Services Administration (HRSA-19-095) funded this project. Funding was used for project training, implementation and QI, but not for reporting on the project. The funding agency was not involved in 1) designing or conducting the study; 2) collecting, managing, analyzing, or interpretating the data; 3) preparing, reviewing, or approving the manuscript; or 4) deciding to submit the manuscript for publication.

## RESULTS

### Program Information and Demographics

The telemedicine pilot program was initiated on September 10, 2020. There were 58 cases for which the telemedicine platform was used. Quality improvement data for patients with chest pain or for whom a 12-lead ECG had been obtained were analyzed 12 months prior to telemedicine implementation (September 9, 2019–September 10, 2020), and 15 months after implementation (September 11, 2020– December 5, 2021). During the pre-implementation period, the EMS agency had a total of 326 medical calls. During the post-implementation period, there were 411 calls. There were 38 chest pain cases in the pre-implementation QI dataset and 38 cases during the post-implementation period.

After implementation of the telemedicine program, 24 (63%) patients eligible for telemedicine consultation received telemedicine services in real time, compared to two (5%) patients in the pre-implementation period. Pre-implementation patients received real-time online medical direction by either phone or radio. During the entire 15-month project, paramedics/EMTs used the telemedicine system 58 times. Outside the primary use for patients with chest pain, other uses included various complicated medical and medicolegal situations in which the paramedic/EMT would have normally called by phone or radio for online medical direction.

The demographic and quality benchmark data collected as part of the pilot program is illustrated in Table 2. The pre-implementation chest pain patient mean age was slightly lower at 72 years old (interquartile range [IQR] 55–80) than the post-implementation patients mean of 75 (IQR 55–80). The percent of non-White ethnicity (identified by paramedic) increased from 11% in the pre-implementaion cohort to 24% in the post-implementation cohort. The EMS agency medication administration rates and ECG acquisition rates were essentially the same.

**Table 2.** Demographics and chest pain call quality improvement benchmark performance.

	Pre-implementation	Post-implementation	*P*-value (p) or odds ratio (OR) with 95% confidence interval (95% CI)
Number of 911 calls	38	38	
Mean age	72	75	*P* = 0.34
Gender, female	39% (n = 15)	53% (n = 20)	1.70 (0.69–4.24)
Ethnicity			
% White	82% (n = 31)	74% (n = 28)	0.63 (0.21–1.89)
% Non-White	11% (n = 4)	24% (n = 9)	4.02 (1.09–14.84)
% Unknown	8% (n = 3)	3% (n = 1)	0.31 (0.03–3.09)
Benchmark performance			
3-lead acquired	100% (n = 38)	97% (n = 37)	0.33 (0.01–8.23)
12-lead acquired	97% (n = 37)	97% (n = 37)	1.00 (0.06–16.59)
Aspirin administered	45% (n = 17)	42% (n = 16)	1.11 (0.45–2.76)
Nitroglycerin administered	21% (n = 11)	18% (n = 7)	1.80 (0.61–5.30)
O_2_ administered	37% (n = 14)	37% (n = 14)	1.00 (0.40–2.48)
Primary impression			
Chest pain	76% (n = 29)	79% (n = 30)	0.91 (0.29–2.82)
Palpitations	11% (n= 4)	13% (n = 5)	1.29 (0.32–5.22)
Difficulty breathing	5% (n = 2)	3% (n = 1)	2.06 (0.18–23.68)
Hypertension	5% (n = 2)	3% (n = 1)	2.06 (0.18–23.68)
Abdominal pain	3% (n = 1)	3% (n = 1)	1.00 (0.06–16.59)

*O*_2_, oxygen.

### Primary Outcome

Overall, there was a slight reduction in BLS transports and slight increase in ALS transports, although these did not achieve statistical significance (see Table 3). There was also a non-significant increase in HEMS transport rates in the implementation group. Lastly, there was a non-significant reduction in patient refusal.

**Table 3.** Disposition of patients included in the chest pain quality improvement dataset.

Mode of transport	Pre-implementation percent (n)	Post-implementation percent (n)	Odds ratio	95%CI	*P*-value
BLS transports	8% (3)	5% (2)	0.65	0.10–4.12	0.65
ALS transports	42% (16)	45% (17)	1.11	0.45–2.76	0.82
HEMS transport	16% (6)	29% (11)	2.17	0.71–6.65	0.17
Refusal	24% (13)	21% (8)	0.51	0.18–1.43	0.20

*BLS*, Basic Life Support; *ALS*, Advanced Life Support; *HEMS*, helicopter emergency medical service; *CI*, confidence interval.

### Secondary Outcome – EMS System Performance

The EMS agency response and transport times did not change significantly following implementation (see Table 4). Median out-of-service time was 127 minutes (IQR 49–172) before and 95 minutes (IQR 52–159) after implementation. This total out-of-service interval included a median response time of 11 minutes (IRQ 1–19) in the pre-implementation cohort and 13 minutes (IQR 7–16) post-implementation. Median on-scene time was 27 minutes (IQR 21–61) in the pre-implementation group and 28 minutes (IQR 20–29) post-implementation. Among those patients who were transported, median transport time was 124 minutes (IQR 49–172) before and 90 minutes (IQR 14–141) after implementation.

**Table 4.** Emergency medical services system utilization times.

	Pre-implementation	Post-implementation	*P*-value
Response time (minutes)			
Mean	11	11	0.96
90^th^ percentile	24	20	
On-scene time (minutes)			
Mean	47	33	0.07
90^th^ percentile	101	61	
Transport time (minutes)			
Mean	114	98	0.54
90^th^ percentile	156	178	
Total EMS call time (minutes)			
Mean	113	105	0.61
90^th^ percentile	194	196	

*EMS*, emergency medical services.

### Secondary Outcome – Performance of Telemedicine Platform

Following implementation, QI data were collected from the caller/call recipients for 35 of 58 (60%) calls. In post-call surveys completed by paramedics/EMTs, the call quality was noted to be “good” or “fair” in 86% of the calls. The EMS physicians judged 98% of the calls to be “good” or “fair.” Connectivity issues were identified as concerns more often by paramedics/EMTs than by EMS physicians (see Table 5).

**Table 5.** Emergency medical services responder and physician subjective evaluation of telemedicine call quality.

	Paramedics/EMTs, percent (number)	EMS physicians, percent (number)
Completed surveys	62% (36)	60% (35)
Call quality		
Good	64% (23)	89% (31)
Fair	22% (8)	9% (3)
Poor	14% (5)	3% (1)
Connectivity issue		
Any issue	64% (23)	14% (5)
Poor cell signal	6% (2)	3% (1)
Lagging video	6% (2)	6% (2)
Poor sound	3% (1)	0% (0)
Missed call	6% (2)	6% (2)
Other	19% (7)	0% (0)

*EMS*, emergency medical service; *EMT*, emergency medical technician.

## DISCUSSION

While larger EMS agencies have adopted telemedicine services to improve the breadth of services provided to their communities (eg, MDAlly, ETHAN project),^13^ ^–^ ^15^ rural EMS agencies have been slower to adopt these services due to limited access to high-quality telecommunication systems and low utilization rates.^16^ ^,^ ^17^ Increasingly, platforms such as AT&T’s “FirstNet” and Verizon’s “Frontline” have become more accessible to rural EMS systems.^18^ ^,^ ^19^ These systems provide high-speed data services and prioritize first-responder communications in times of high system usage.^17^ ^,^ ^20^ ^,^ ^21^ With these tools now available to rural EMS agencies, it is important to evaluate the impact that telemedicine programs have on these systems.

In this retrospective review, a rural EMS telemedicine system was used 58 times during the 15-month pilot project for patients with a variety of out-of-hospital medical emergencies. Overall, the telemedicine system functioned well with 89% of EMS physicians and 65% of paramedics/EMTs rating the technical quality of the telemedicine encounter as “good.” Of note, the use of any form of online medical direction increased dramatically to 63% in the post-implementation phase in comparison to 5% in the pre-implementation phase.

Our primary goal in this study was to evaluate the impact of telemedicine service on the care of patients with chest pain and determine whether telemedicine might change mode of transport (BLS vs ALS vs HEMS) to the closest hospital with percutaneous intervention capabilities. The ultimate goal was to allow ALS responders to stay in the community and increase the amount of time ALS service was available. In this retrospective analysis, we found no statistically significant difference demonstrated in the BLS/ALS transport rates after the telemedicine program was implemented despite a goal of increasing BLS transports. There are several possible reasons that no change in transport rates was observed. Primarily, the small patient-sample size both before and after the intervention limits the ability to detect a change. Moreover, the period following the intervention coincided with the onset of the COVID-19 pandemic, an event that likely increased patient acuity levels as well as the utilization of HEMS transport, given the heightened concern over virus transmission. In this context, ground ambulance transport for long distances may have been considered less safe compared to HEMS, due to the perceived increased risk of COVID-19 exposure, as ground transport times are much longer than HEMS transport times.

During implementation, paramedics/EMTs voiced several concerns about using telemedicine in the rural EMS setting. These included the concern that contacting a physician via the telemedicine platform would increase their on-scene time and overall out-of-service times. In this small cohort, the EMS system on-scene times and out-of-service times were not increased but rather trended toward being shorter. Two factors likely contributed to this finding. First, with multiple responders on scene for most 911 responses, one team member was able to discuss the case with a physician while the rest of the team continued to provide patient care. Second, although not measured as part of this project, telemedicine call duration seemed to be very brief (1–2 minutes) and likely this short encounter did not significantly change a relatively longer EMS on-scene time.

The quality of telemedicine communication was a concern for rural paramedics/EMTs. In this retrospective review we found that self-reported telemedicine system performance was mostly reported as good. Paramedics/EMTs were more likely to have concerns about the telemedicine system than were the EMS physicians. System performance could have been worse in the rural setting due to limited data transfer; in other words, cellular service was more likely to be poor in the rural than urban setting. However, if that had been the case, one would expect both parties to have the same issue. It is also possible that with a large EMS user group and a small EMS physician user group, the EMS physicians were simply more comfortable using the platform and experienced fewer technical issues.

The majority of telemedicine concerns expressed by paramedics/EMTs, and EMS physicians were lagging video and poor cell signal. There were seven instances in which paramedics/EMTs listed “other” issues with the platform, but further information was not available. It would be expected that there would be more telemedicine technical issues on the paramedic/EMT side in rural areas than on the EMS physician side where physician took calls in an urban, academic center.

## LIMITATIONS

Limitations included the data collection and implementation of the telemedicine pilot project at a single EMS agency. Additionally, this analysis relied on retrospective review of data collected for the purpose of QI. These two factors introduce both the strong possibility of observer bias and reporting bias. Also, the small number of encounters limit statistical analysis and significance. It is possible that not all chest pain patients were included in the QI dataset and missing or included patients created an inclusion bias.

The post-intervention study period coincided with the COVID-19 pandemic. The massive psycho-social changes that occurred during this period almost certainly impacted the community in which this pilot program was conducted. Unfortunately, the impact of the pandemic on this dataset is unknown; however, the unfortunate timing likely introduced a confounder into our results. Finally, the EMS agency participating in this project was highly engaged and motivated, as demonstrated by high performance on EMS benchmarks. It is possible that it was difficult to detect a change in system performance due to both the low call volume and the high-quality patient care already provided by the EMS agency.

## CONCLUSION

In this rural EMS system, a telehealth platform was successfully used to connect paramedics/EMTs to board-certified EMS physicians over a 15-month period for 58 patients. Among those patients with chest pain, the use of telemedicine did not result in any change in the rate of ALS transports or increase on-scene times. Overall, paramedics/EMTs and EMS physicians rated the quality of the telemedicine connection as good. Future studies could expand upon this work by exploring larger patient populations and diverse clinical conditions to further establish the efficacy of telemedicine in rural EMS settings. Additionally, examining long-term patient outcomes and cost effectiveness could provide more insight into the sustained impact of telehealth interventions on rural emergency care.
